# Supplementary material for: Time series analysis and mechanistic modelling of heterogeneity and sero-reversion in antibody responses to mild SARS‑CoV-2 infection
Source: eBioMedicine. 2021 Mar 2;65:103259. doi: 10.1016/j.ebiom.2021.103259 (PMC7920816; doi:10.1016/j.ebiom.2021.103259)
Supplement: Supplementary file 1 [file mmc1.pdf]

Supplementary data

Time series analysis and mechanistic modelling of heterogeneity and sero-reversion in antibody responses to mild SARS-CoV-2 infection.

Contents

Supplementary Table 1. Baseline characteristics of anti-S1 seroconverters 2

Supplementary Table 2. Peak anti-S1 response 2

Supplementary Table 3. Peak anti-NP response 2

Supplementary Table 4. Anti-S1 time to sero-reversion.. 2

Supplementary Figure 1. Relationship between antibody levels as measured by Euroimmun and Roche assays, and antibody dilution series. 3

Supplementary Figure 2. Mathematical model simulations of effect of selected parameters in changes to antibody levels. 4

Supplementary Figure 3. Individual participant level serology data. 5

Supplementary Figure 4. Association of seropositivity to SARS-CoV-2 with demographic, clinical and exposure factors. 6

Supplementary Figure 5. Correlation of peak antibody responses in Euroimmun anti-S1 and Roche anti-NP assays.. 7

Supplementary Figure 6. Correlation in antibody levels stratified by time from infection................................. 8

Supplementary Figure 7. Model fit to individual participant data... 9

Supplementary Figure 8. Mathematical modelling of kinetics of circulating anti-S1 and anti-NP antibodies in baseline seronegative participants. 10

Supplementary Table 1. Baseline characteristics of anti-S1 seroconverters

Characteristic	Overall, N = 142	Anti-S1 Seroreversion, N = 32[1]	No anti-S1 seroreversion, N = 110[1]
Age	38 (30, 49)	35 (32, 46)	38 (30, 50)
Male (vs. Female)	49 (35%)	9 (28%)	40 (36%)
BAME (vs. White)	42 (31%)	6 (20%)	36 (34%)
Unknown	7	2	5
Case-defining symptoms (Yes vs. No)	63 (44%)	10 (31%)	53 (48%)
Peak anti-S1	5.19 (2.85, 7.38)	2.30 (1.66, 3.30)	5.79 (3.77, 8.25)
Peak anti-NP	63 (19, 107)	18 (9, 37)	79 (34, 115)
Unknown	7	5	2

[1]Statistics presented: median (IQR); n (%)

Supplementary Table 2. Peak anti-S1 response

	Univariable				Multivariable		
Characteristic	N	Beta	95% CI[1]	p-value	Beta	95% CI[1]	p-value
Age (per year increase)	142	0.06	0.01, 0.10	0.009	0.05	0.01, 0.09	0.021
Male (vs. Female)	142	0.50	-0.51, 1.5	0.3	0.09	-0.93, 1.1	0.9
BAME (vs. White)	135	1.1	0.06, 2.2	0.039	1.00	-0.04, 2.0	0.058
Case-defining symptoms (Yes vs. No)	142	0.78	-0.19, 1.7	0.11	0.82	-0.14, 1.8	0.094

[1]CI = Confidence Interval

Supplementary Table 3. Peak anti-NP response

	Univariable				Multivariable		
Characteristic	N	Beta	95% CI[1]	p-value	Beta	95% CI[1]	p-value
Age (per year increase)	150	0.61	-0.09, 1.3	0.085	0.50	-0.22, 1.2	0.2
Male (vs. Female)	149	2.4	-14, 19	0.8	0.05	-17, 17	>0.9
BAME (vs. White)	144	23	6.0, 40	0.008	22	4.9, 39	0.012
Case-defining symptoms (Yes vs. No)	150	1.5	-14, 17	0.9	-0.79	-16, 15	>0.9

[1]CI = Confidence Interval

Supplementary Table 4. Anti-S1 time to sero-reversion

	Univariable				Multivariable		
Characteristic	N	HR[1]	95% CI[1]	p-value	HR[1]	95% CI[1]	p-value
Age (per year increase)	142	0.98	0.95, 1.01	0.2	1.01	0.97, 1.05	0.6
Male (vs. Female)	142	0.93	0.43, 2.01	0.9	1.06	0.46, 2.45	0.9
BAME (vs. White)	135	0.58	0.24, 1.43	0.2	1.03	0.41, 2.64	>0.9
Case-defining symptoms (Yes vs. No)	142	0.65	0.30, 1.38	0.3	0.66	0.30, 1.46	0.3
Peak anti-S1 (per unit increase)	142	0.41	0.30, 0.57	<0.001	0.42	0.30, 0.58	<0.001

[1]HR = Hazard Ratio, CI = Confidence Interval

Supplementary Figure 1. Relationship between antibody levels as measured by Euroimmun and Roche assays, and antibody dilution series.

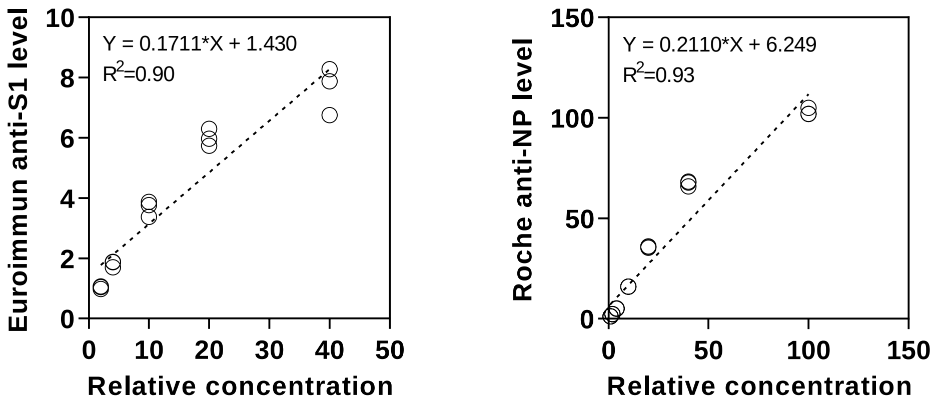

The measurement of antibody levels for each assay is shown across a range of serial dilutions.

Supplementary Figure 2. Mathematical model simulations of effect of selected parameters in changes to antibody levels.

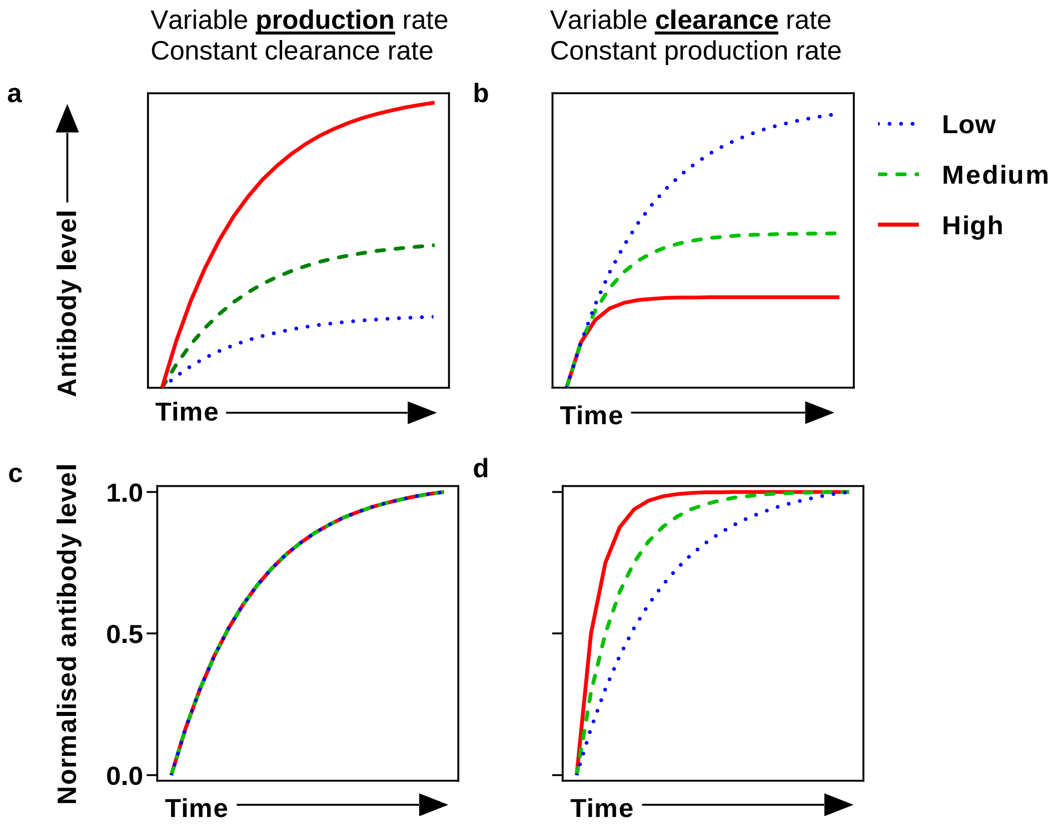

Increasing rates of antibody production at a constant rate of clearance leads higher antibody levels **(a)** but identical temporal profiles of antibody levels normalised to the peak **(c)**. Increasing rates of clearance at a constant rate of production lead to lower antibody levels **(b)**, but shorter time to peak antibody levels **(d)**.

Supplementary Figure 3. Individual participant level serology data.

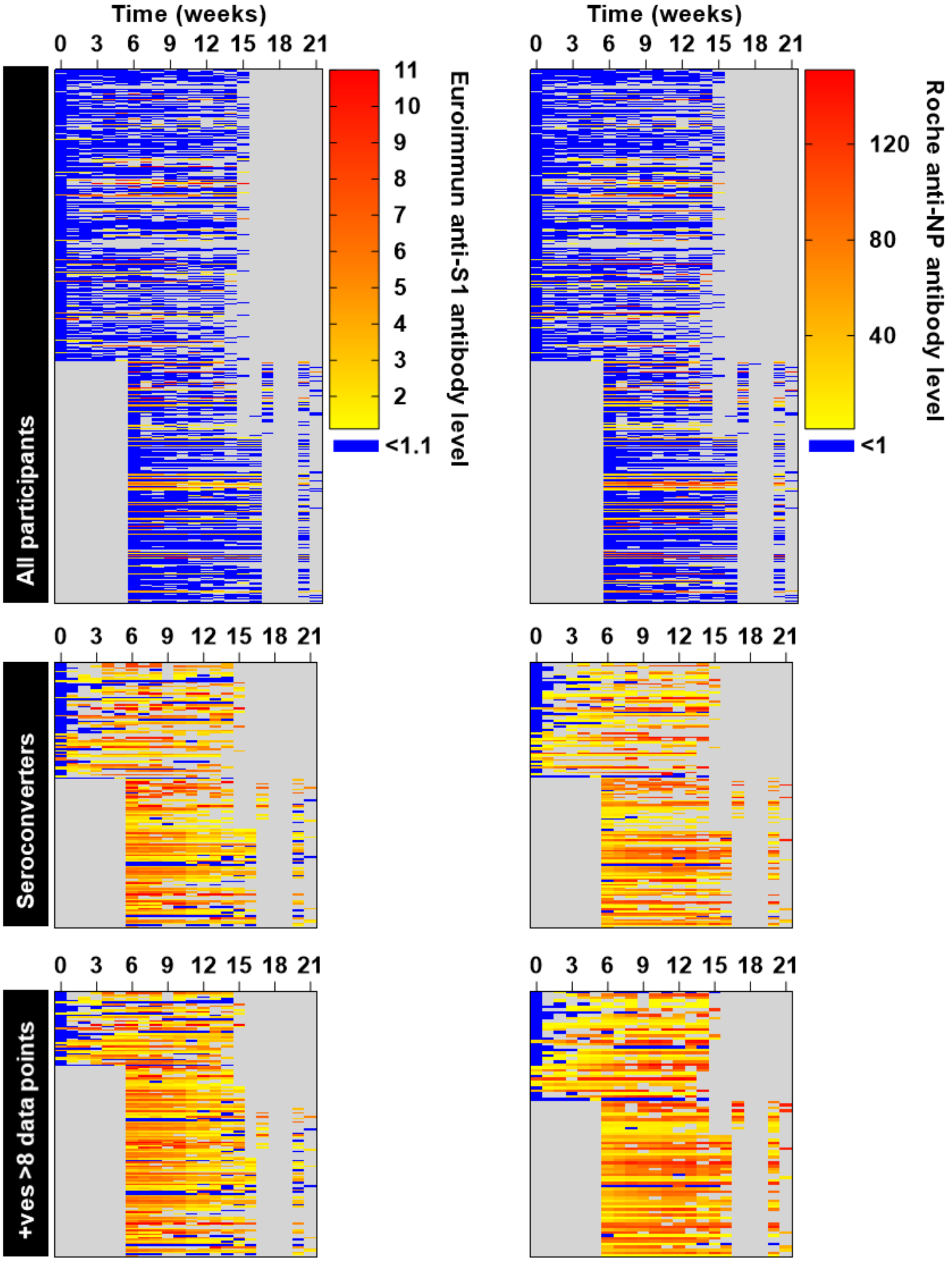

Heatmaps show antibody levels in each assay and missingness (grey) for individual participants over 0-21 weeks, for all participants (top row), all participants who were seropositive by at least one assay at any time point (middle row), and all such participants who had >8 data points available (bottom row), used for the mathematical modelling.

Supplementary Figure 4. Association of seropositivity to SARS-CoV-2 with demographic, clinical and exposure factors.

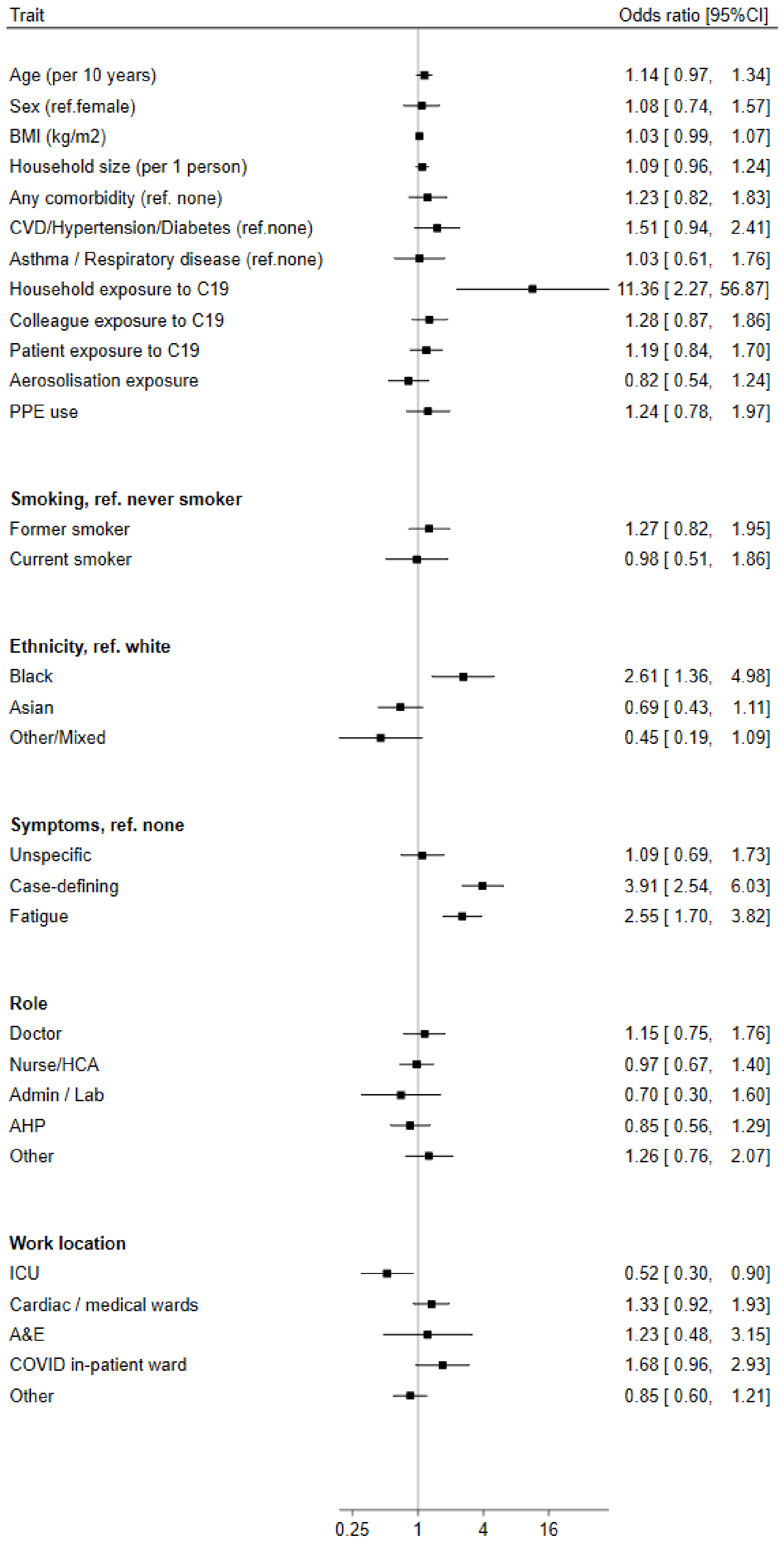

Odds ratios of selected demographic variables for seropositivity defined a participants who were seropositive at any time point using either assay across the study period

Supplementary Figure 5. Correlation of peak antibody responses in Euroimmun anti-S1 and Roche anti-NP assays

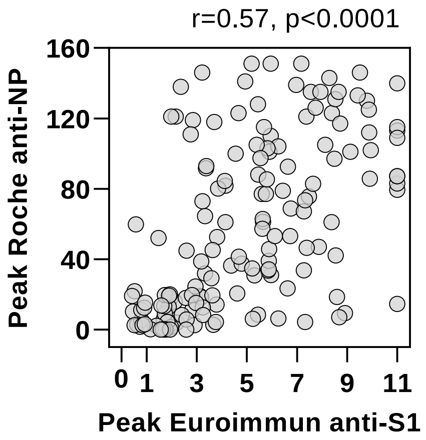

Peak antibody levels in each assay are shown for all participants who were seropositive by either assay at any time point, with Spearman rank correlation.

Supplementary Figure 6. Correlation in antibody levels stratified by time from infection.

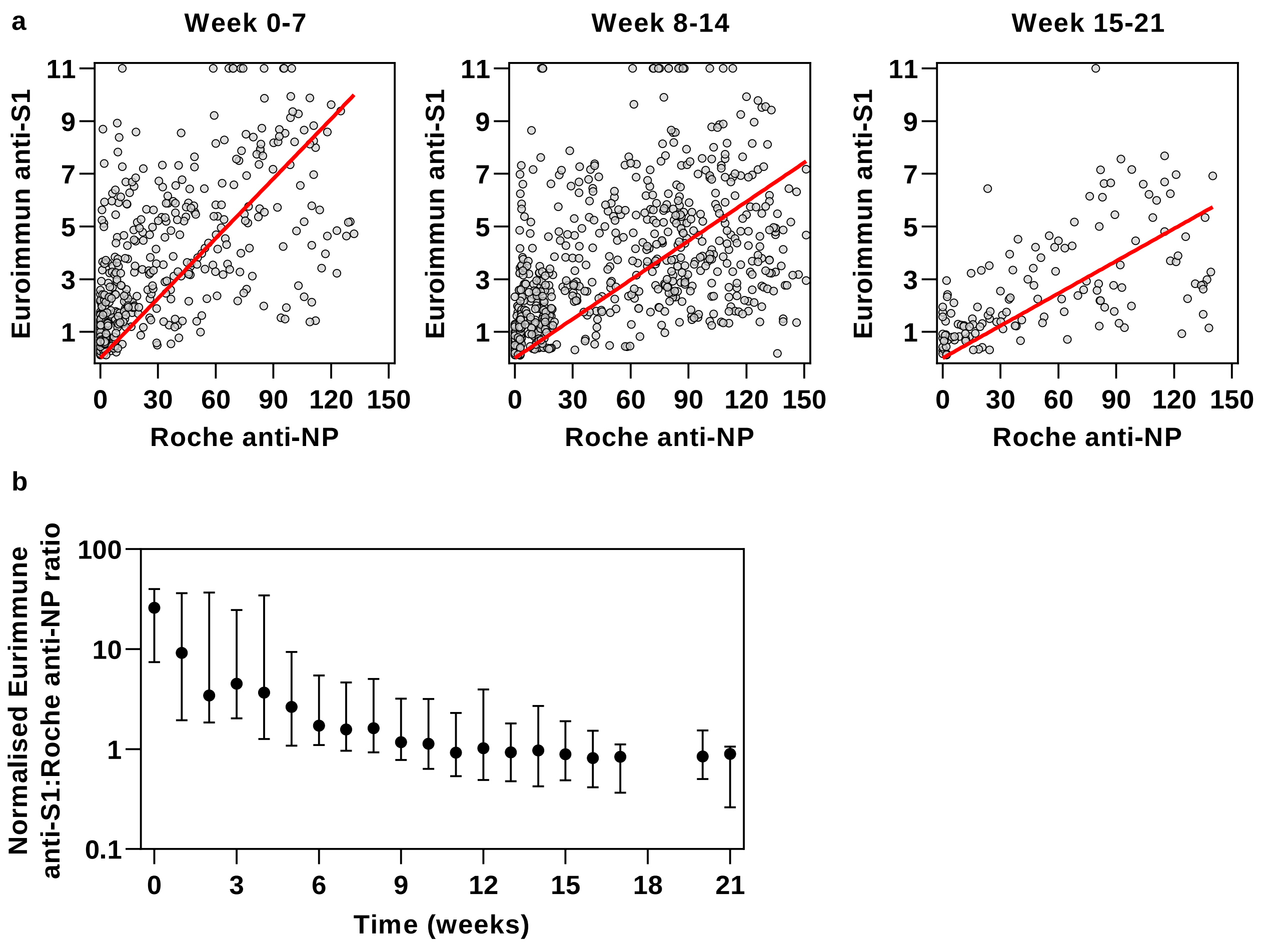

(a) For all participants who were seropositive by either assay at any time point, antibody levels from each assay are compared in three time intervals weeks 0-7 (left), weeks 8-14 (centre) and weeks 15-21 (right). Red line shows the linear regression. **(b)** The normalised (by maximum value for each assay) ratio of Euroimmun antiS1 to Roche anti-NP by study week, showing median and interquartile range.

Supplementary Figure 7. Model fit to individual participant data

A

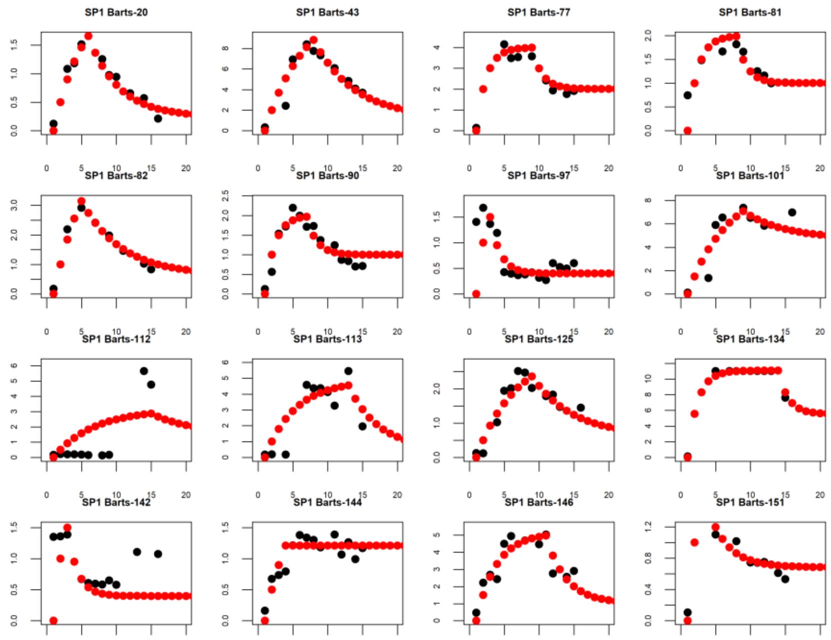

b

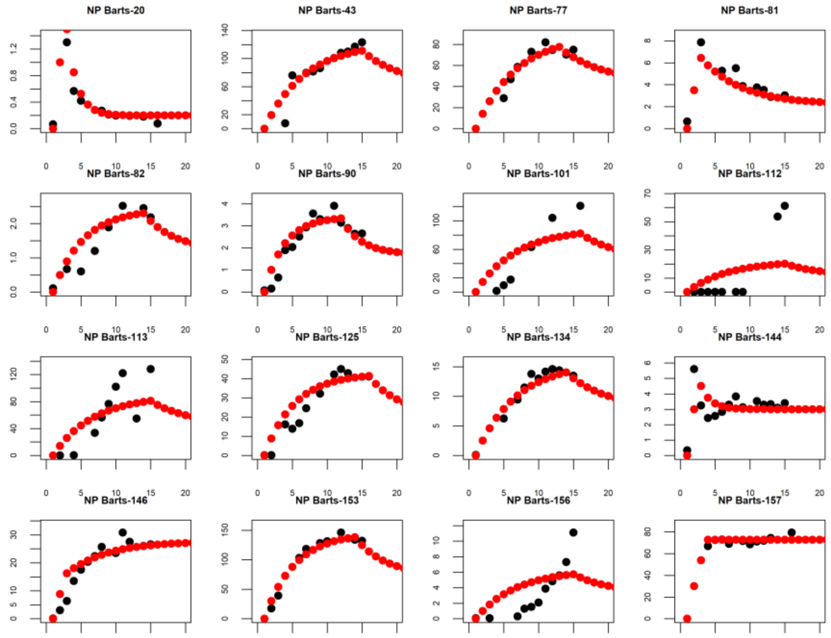

Representative models (red) fit to serology data (black) for **(a)** Euroimmun anti-S1 measurments, and **(b)** Roche anti-NP measurements in 16 randomly selected individuals from participants who were seropositive with either assay at any time point, and had >8 serology data points available.

Supplementary Figure 8. Mathematical modelling of kinetics of circulating anti-S1 and anti-NP antibodies in baseline seronegative participants.

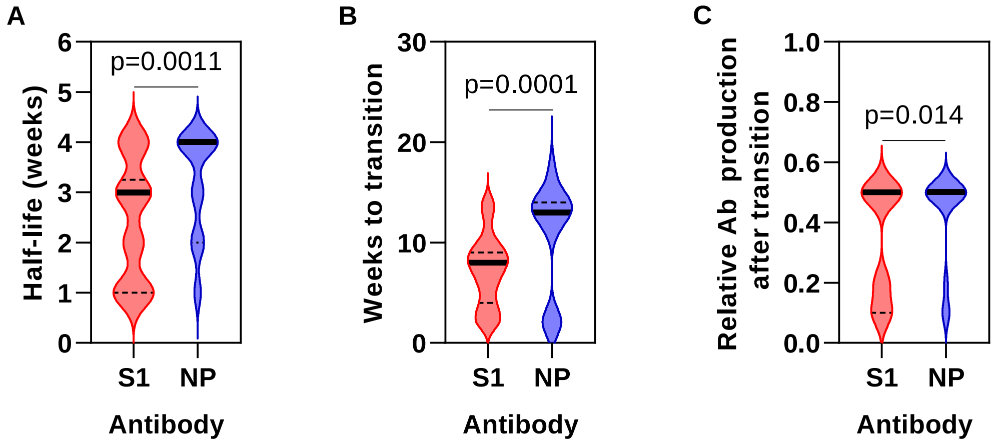

Best fit model parameters for **(a)** half-life of antibody clearance, **(b)** time to transition point of lower antibody production **(c)** and relative reduction in antibody production following this transition point, for individual baseline seronegative participants who subsequently seroconvert (N=50 for anti-S1 serology, and 44 for anti-NP serology; p values derived from 2-tail Mann Whitney tests).
